# Radiologic-pathologic correlation in breast cancer: do MRI biomarkers correlate with pathologic features and molecular subtypes?

**DOI:** 10.1186/s41747-022-00289-7

**Published:** 2022-08-08

**Authors:** Francesca Galati, Veronica Rizzo, Giuliana Moffa, Claudia Caramanico, Endi Kripa, Bruna Cerbelli, Giulia D’Amati, Federica Pediconi

**Affiliations:** Department of Radiological, Oncological and Pathological Sciences, Sapienza University of Rome, 00161 Rome, Italy

**Keywords:** Biomarkers, Breast neoplasms, Multiparametric magnetic resonance imaging, Pathology (molecular), Precision medicine

## Abstract

**Background:**

Breast cancer (BC) includes different pathological and molecular subtypes. This study aimed to investigate whether multiparametric magnetic resonance imaging (mpMRI) could reliably predict the molecular status of BC, comparing mpMRI features with pathological and immunohistochemical results.

**Methods:**

This retrospective study included 156 patients with an ultrasound-guided biopsy-proven BC, who underwent breast mpMRI (including diffusion-weighted imaging) on a 3-T scanner from 2017 to 2020. Histopathological analyses were performed on the surgical specimens. Kolmogorov–Smirnov Z, *χ*^2^, and univariate and multivariate logistic regression analyses were performed.

**Results:**

Fifteen patients were affected with ductal carcinoma *in situ*, 122 by invasive carcinoma of no special type, and 19 with invasive lobular carcinoma. Out of a total of 141 invasive cancers, 45 were luminal A-like, 54 luminal B-like, 5 human epidermal growth factor receptor 2 (HER2) positive, and 37 triple negative. The regression analyses showed that size < 2 cm predicted luminal A-like status (*p* = 0.025), while rim enhancement (*p* < 0.001), intralesional necrosis (*p* = 0.001), peritumoural oedema (*p* < 0.001), and axillary adenopathies (*p* = 0.012) were negative predictors. Oppositely, round shape (*p* = 0.001), rim enhancement (*p* < 0.001), intralesional necrosis (*p* < 0.001), and peritumoural oedema (*p* < 0.001) predicted triple-negative status.

**Conclusions:**

mpMRI has been confirmed to be a valid noninvasive predictor of BC subtypes, especially luminal A and triple negative. Considering the central role of pathology in BC diagnosis and immunohistochemical profiling in the current precision medicine era, a detailed radiologic-pathologic correlation seems vital to properly evaluate BC.

## Key points

- Early discrimination of the breast cancer subtypes is a key step in treatment planning.
- Molecular subtypes are routinely determined by percutaneous biopsy and immunohistochemical surrogate analysis.
- Multiparametric magnetic resonance imaging could be a valid noninvasive tool for characterising different subtypes of breast cancer.

## Background

Tumour heterogeneity is a well-known characteristic of breast cancer (BC) [1]. With the spread of technologies of molecular biology and the growing knowledge of the biological processes underlying the development of BC, the importance of biomarkers has progressively grown.

Three molecular biomarkers, namely oestrogen receptor, progesterone receptor and human epidermal growth factor receptor 2 (HER2), along with the proliferation index (Ki-67), are currently used, together with traditional parameters (*e.g.,* tumour size, histological grade, and lymph node involvement), in the routine clinical management of BC patients to choose the appropriate treatment and to predict prognosis and tumour response to therapy [1–3]. The molecular subtypes of BC are nowadays routinely determined using immunohistochemical surrogates [4]. According to immunohistochemistry, five subtypes of breast cancer were identified: luminal A-like, luminal B-like HER2 negative, luminal B-like HER2 positive, HER2 positive, and triple negative [5].

Considering the more aggressive clinical behaviour and worse prognosis of triple-negative and HER2 positive BCs as compared to the luminal-like ones, the early discrimination of these molecular subtypes with noninvasive breast imaging may integrate the histopathological results, facilitate the interpretation of histological examination and, ultimately, may be useful to clinicians for planning the most suitable patient management. For this reason, multidisciplinary team meetings (including breast radiologists, pathologists, surgeons, and oncologists) are nowadays considered the best practice in BC management.

Multiparametric magnetic resonance imaging (mpMRI) combines morphologic (T2-weighted sequences), functional (diffusion-weighted imaging, DWI), and kinetic (contrast-enhanced sequences) data, in the attempt to provide information about the development of BC and the response to therapy, among others [6]. Previous studies have suggested that certain BC subtypes show peculiar features at mpMRI, implying that this technique could differentiate these subtypes noninvasively [7–12].

T2-weighted sequences increase breast MRI specificity, since the great majority of BCs has intermediate to low T2 signal intensity [13, 14], and allow the detection of lesion-associated features, such as intralesional necrosis (commonly accompanying the triple-negative aggressive phenotype) and peritumoural oedema, a suggested indicator of poor prognosis [8, 10]. DWI measures the water diffusivity of the tissues under examination and represents a valuable tool to distinguish benign from malignant breast lesions [15], showing higher specificity than contrast-enhanced sequences [6, 16]. Moreover, DWI has shown the potential to predict lesions aggressiveness, in terms of tumour receptor status [7, 11, 12] and nuclear grading [10, 11, 17]. T1-weighted contrast-enhanced sequences are a key tool in breast mpMRI, having the highest sensitivity and a very good specificity for BC identification. The development of new, abnormal and highly permeable blood vessels (neoangiogenesis) is a fundamental step in cancer growth [18] and represents the basis of BC detection on contrast-enhanced images.

The aim of this study was to verify whether diagnostic breast mpMRI could be used to reliably predict the molecular status of BC in terms of radiologic-pathologic correlation. Accordingly, we investigated the mpMRI features of BC subtypes at 3 T and compared them with immunohistochemical and pathological results.

## Methods

### Study population

This study was conducted according to Good Clinical Practice guidelines and obtained the approval of our institutional review board (no. 0525032019, 25 March 2019). The requirement for informed consent was waived because of the retrospective nature of the study.

From January 2017 to January 2020, patients with a newly diagnosed ultrasound-guided biopsy-proven BC who underwent breast mpMRI at our institution at the time of diagnosis (according to the multidisciplinary team indications) were considered for this study (*n* = 364). Ultrasound-guided core needle biopsies were performed by two experienced breast interventional radiologists using a 12 MHz linear probe (SSA-700A; Toshiba, Tokyo, Japan/Affiniti70G; Philips, Amsterdam, Netherlands) and a 14-gauge semiautomatic biopsy needle (Precisa; HS Hospital Service S.p.A., Aprilia, Italy). Definitive breast surgery (including lumpectomy, quadrantectomy and mono- or bilateral mastectomy) was performed within 1 month from mpMRI in all patients.

Exclusion criteria included the following: incomplete MRI examination (*n* = 14); previous BC or recurrent disease (*n* = 49); ongoing neoadjuvant chemotherapy or other cancer therapy (*n* = 45); breast implants (*n* = 32); core needle biopsy performed less than 14 days before MRI, to eliminate possible bias due to the diagnostic procedure (*n* = 28); and histologic studies or surgery performed in another Institution (*n* = 40). Accordingly, 156 patients were included in the study.

Clinical and histopathological data were retrieved from our institutional database (Excel 2011, Microsoft Corporation, Redmond, WA, USA).

### mpMRI technique

All MRI examinations were performed on a 3-T magnet (Discovery MR 750; GE Healthcare, Chicago, IL, USA) using a dedicated 8-channel breast coil compatible with parallel imaging and patients in a prone position. The mpMRI examination protocol used for the study included the following:

- Axial unenhanced two-dimensional fast spin-echo T2-weighted fat-suppressed sequences (repetition time [RT] 9,000–11,000 ms, echo time [ET] 119–120 ms, matrix 512 × 224, slice thickness 3–5 mm, field of view [FOV] 350 × 350 mm, number of excitations [NEX] 1, scan time 130 s)
- Axial unenhanced two-dimensional DWI echo-planar sequence (RT 4,983–5,314 ms, ET 58 ms, matrix 150 × 150, slice thickness 3 − 5 mm, FOV 350 × 350 mm, *NEX* = 2–4, scan time 230 s)
- Axial dynamic three-dimensional spoiled gradient-echo T1-weighted fat-suppressed (VIBRANT) sequences (flip angle 15°, RT 8 ms, ET 4 ms, matrix 512 × 256, slice thickness 1.40 mm, FOV 380 × 380 mm, NEX 1, total scan time 120 s) or axial dynamic dual-echo three-dimensional spoiled gradient-recalled T1-weighted fat-suppressed (DISCO) sequences (flip angle 15°, RT 4 ms, ET 2 ms, bandwidth 166.67 kHz, matrix 320 × 320, slice thickness 1.40 mm, FOV 340 × 340 mm, NEX 1, total scan time 360 s), VIBRANT sequences were performed before and four times after contrast agent administration and DISCO sequences before and nine times after.
- And sagittal three-dimensional spoiled GE post-contrast T1-weighted sequence (flip angle 15°, RT 4 ms, ET 2 ms, bandwidth 142.86 kHz, matrix 224 × 320, slice thickness 4 mm, FOV 300 × 300 mm, NEX 1, total scan time 134 s)

Fat suppression of T2-weighted sequences was based on a 3-point Dixon technique (IDEAL), whereas fat suppression of T1-weighted sequences was obtained using a 2-point Dixon fat–water reconstruction algorithm. DWI echo-planar sequences included *b*-values of 0, 500 and 1,000 s/mm^2^, and the corresponding apparent diffusion coefficient (ADC) maps were calculated automatically. Post-contrast T1-weighted images were acquired after the administration of 0.1 mmol/kg (0.2 mL/kg) of a gadolinium-based contrast agent (Gadoteridol, ProHance; Bracco Imaging Italia S.r.l., Milano, Italy) at a rate of 3 mL/s. Gadoteridol was power injected through a peripheral venous access (22 Gauge) and followed by a 20-mL saline flush at the same rate. Post-processing subtraction images were obtained for the dynamic series of all examinations. Imaging of premenopausal women was performed between the 7th and the 14th day of the menstrual cycle, according to current guidelines [19].

### mpMRI evaluation

MRI datasets were evaluated retrospectively by two experienced breast radiologists (with 18 and 9 years of experience, respectively), in consensus. The readers were blinded to clinical and histopathological information. The evaluation was performed using all the images available, and MRI suspicious findings were classified according to the 2013 American College of Radiology Breast Imaging Reporting and Data System lexicon [20]. All the lesions were measured, and maximum size in mm was reported.

T2-weighted signal intensity of each lesion was evaluated visually and classified as hypointense (lower intensity than the surrounding glandular tissue), isointense (same intensity), and hyperintense (higher intensity), on the basis of the predominant signal intensity of the lesion. Moreover, unenhanced fat-suppressed T2-weighted images were used to evaluate the possible presence of intralesional necrosis and perilesional oedema, assessed visually as areas of high signal intensity (as high as that of water) within or around the lesion.

DWI signal intensity was evaluated qualitatively on high *b*-value images (*b* = 1,000 s/mm^2^). ADC values of DWI hyperintense lesions were obtained drawing manually a two-dimensional region of interest in the centre of the area of restricted diffusion on ADC maps. Considering that a threshold of 1 × 10^-3^ mm^2^/s has been recommended for distinguishing malignant from benign breast lesions, ADC values were classified in very low (0.0–0.9 × 10^−3^ mm^2^/s), low (1.0–1.3 × 10^-3^ mm^2^/s), and intermediate (1.4–1.8 × 10^-3^ mm^2^/s), even if there are no standardised cutoffs yet established [15, 21].

The presence of axillary lymphadenopathies (characterised by size > 1 cm, round shape, loss of the fatty hilum, and cortical thickening) was assessed on post-contrast fat-suppressed T1-weighted sequences.

### Histopathological analysis

All the surgical specimens were sent to the Department of Pathology of our institution and evaluated according to standardised protocols by two experienced pathologists. The samples were fixed in 10% formalin for 12–24 h, processed to obtain paraffin blocks and subsequently cut in 5-µm-thick slices and stained with haematoxylin–eosin.

Tumours were classified following the World Health Organization classification [22] and graded according to the Nottingham Histologic Score in low (G1), intermediate (G2), and high grade (G3) of malignancy.

The immunohistochemical analysis was carried out using mouse monoclonal antibodies anti-ER alpha (6F11; Novocastra Laboratories Ltd., Newcastle upon Tyne, UK) and anti-PgR (PgR-312; Novocastra Laboratories Ltd., Newcastle upon Tyne, UK). HER2 evaluation was performed using a semiquantitative immunohistochemical assay (HercepTest; DakoAgilent, Santa Clara, CA, USA). The intensity of HER2 membrane staining was scored as 0, 1 + , 2 +, or 3 + . In case of equivocal result (2 +), fluorescence *in situ* hybridisation for HER2 gene amplification was carried out, according to the American Society of Clinical Oncology/College of American Pathologists guidelines [23]. The proliferation index was determined using anti-Ki-67 monoclonal antibody MM1 (Novocastra Laboratories Ltd., Newcastle upon Tyne, UK), and the Ki-67 value was expressed as the percentage of tumour cells showing nuclear staining. According to immunohistochemical features, tumours were classified as luminal A-like, luminal B-like HER2 negative, luminal B-like HER2 positive, HER2 positive and triple-negative, following the St. Gallen Consensus Conference classification [5].

### Statistical analysis

IBM SPSS Statistics v.25 (Chicago, IL, USA) was used to perform statistical analyses, *p*-values < 0.05 being considered significant. The Kolmogorov–Smirnov *Z*-test was performed to assess the normality of the distribution for the continuous variables tested. Continuous normal variables were expressed as mean ± standard deviation, while continuous nonnormal variables were expressed as median and range. Categorical variables were compared using the *χ*^2^ test. The Bonferroni correction was used for post hoc *χ*^2^ analysis. Univariate and multivariate logistic regression analyses were performed to identify the predicting value of imaging-derived features associated with the different molecular subtypes of BC. All variables with a *p*-value < 0.05 at univariate analysis were included in the multivariate analysis.

## Results

### Study population

A total of 156 patients were included in this study, 15 were affected by ductal carcinoma *in situ* (DCIS), 122 by invasive carcinoma of no special type (NST) and 19 by invasive lobular carcinoma (ILC). The 141 invasive BCs detected at histological evaluation were categorised according to the St. Gallen classification as luminal A-like (*n* = 45), luminal B-like (HER2 positive and HER2 negative) (*n* = 54), HER2 positive (*n* = 5) and triple negative (*n* = 37). Patients’ mean age was 53.8 (standard deviation 10.7). Median size of BCs at MRI was 20 mm (range 6–100 mm).

### Invasive carcinomas and DCIS

**Fig. 1:**
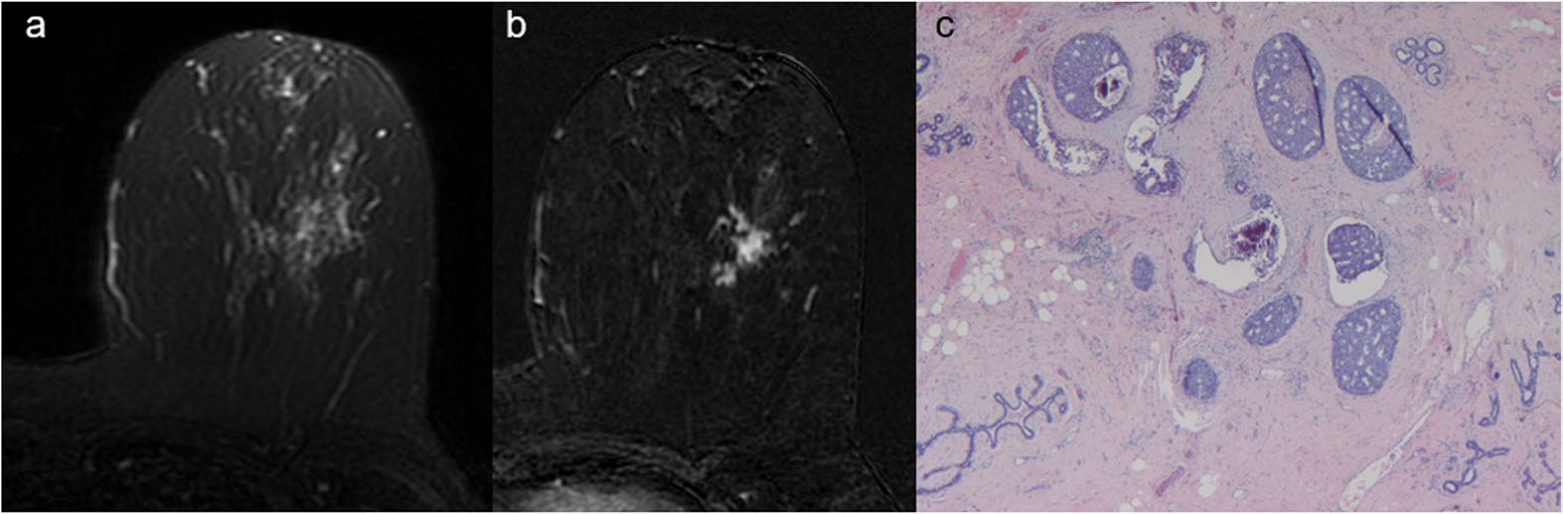
A 59-year-old woman with ductal carcinoma *in situ* of the left breast. **a** Axial fat-suppressed T2-weighted image shows a 21-mm isointense lesion in the lower outer quadrant of the left breast. **b** Axial post-contrast T1-weighted subtracted images show a corresponding regional, clumped non-mass enhancement. **c** Histological examination confirms the suspicion of *in situ *carcinoma highlighting several intraductal calcifications

On MRI examination, the vast majority of invasive lesions appeared as mass enhancements 80.8%), while DCISs appeared as non-mass enhancements (86.7%) (Fig. 1). Statistical analysis demonstrated a significant association between invasive NST carcinoma and mass enhancement, intralesional necrosis, axillary adenopathy and multifocal extension of disease. We also observed a significant association between DCIS and isointensity on T2-weighted images, non-mass enhancement and the absence of central necrosis and of axillary adenopathy. No association was found between ILC and MRI features. Further details are shown in Table 1.

**Table 1:** Association between histological subtypes of breast cancer

		**DCIS**	**χ2 analysis** ***p*-value**	**Invasive carcinoma** no special type	**χ2 analysis *p*-value**	**ILC**	**χ2 analysis** ***p*-value**
No. of patients		15		122		19	
Tumour size	Median (mm) Range (mm)	38 7–72		20 6–90		26 8–100	
≥ 2 cm		10/15	0.429	65/122	0.071	14/19	0.118
T2 intensity	Hypointensity Isointensity Hyperintensity	0/15 12/15 3/15	< 0.001	55/122 30/122 37/122	0.015^a^	9/19 5/19 5/19	0.833
Mass enhancement		2/15	< 0.001	102/122	< 0.001	12/19	0.233
Rim enhancement		0/2	0.292	39/102	0.079	2/12	0.153
Intralesional necrosis		0/15	0.001	53/122	0.035	8/19	0.775
Perilesional oedema		6/15	0.358	67/122	0.085	7/19	0.179
Axillary adenopathy		0/15	0.003	48/122	0.007	5/19	0.452
Intensity-to-time curve	Type I Type II Type III	1/15 5/15 9/15	0.801	10/122 31/122 81/122	0.877	2/19 5/19 12/19	0.932
ADC value	Very low Low Intermediate	3/10 6/10 1/10	0.055	70/111 39/111 2/111	0.144	7/14 7/14 0/14	0.575
Extent of disease	Unifocal Multifocal Multicentric	7/15 0/15 8/15	0.018 ^a^	55/122 39/122 28/122	0.02	9/19 2/19 8/19	0.170

^a^Bonferroni’s post hoc analysis found no significance.  *ADC* Apparent diffusion coefficient, *DCIS* Ductal carcinoma in situ, *ILC* Invasive lobular carcinoma

### Grading

A total of 63 lesions were characterised by a low or intermediate grade of differentiation (G1 or G2) and 73 by a high grade of differentiation (G3). The remaining 20 lesions were not classifiable at histopathological analysis. Statistical analyses found a significant association between G3 tumours and rim enhancement, intralesional necrosis, peritumoural oedema, axillary adenopathy, and multifocal disease. The univariate analysis proved that rim enhancement, intralesional necrosis, peritumoural oedema, the presence of malignant axillary lymph nodes, and very low ADC values were predictors of high grade of malignancy. The multivariate analysis confirmed rim enhancement as the only independent predictor for high grade of malignancy (Table 2).

**Table 2:** Association between breast cancer grading (high grade) and magnetic resonance imaging features

		**High grade**	**χ2 analysis** ***p*-value**	**Univariate analysis**		**Multivariate analysis**	
				**OR (CI 95%)**^b^	***p*-value**	**OR (CI 95%)**^b^	***p*-value**
**No. of patients**		73					
**Tumour size**	Median (mm) Range (mm)	52 34–75					
** ≥ 2 cm**		44/73	0.097	1.779 (0.899–3.518)	0.098		
**T2 intensity**	Hypointensity Isointensity Hyperintensity	35/73 16/73 22/73	0.047^a^	1.842 (0.918–3.697) 0.399 (0.189–0.844) 1.267 (0.595–2.699)	0.086 0.016 0.539	0.385 (0.124–1.193)	0.098
**Mass enhancement**		55/73	0.921	1.040 (0.478–2.264)	0.921		
**Rim enhancement**		25/55	0.002	4.062 (1.607–10.271)	0.003	3.689 (1.011–3.467)	0.048
**Intralesional necrosis**		33/73	0.028	2.232 (1.084–4.597)	0.029	0.651 (0.193–2.195)	0.489
**Perilesional oedema**		45/73	0.001	3.214 (1.588–6.505)	0.001	2.105 (0.729–6.078)	0.169
**Axillary adenopathy**		32/73	0.008	2.732 (1.287–5.799)	0.009	2.241 (0.769–6.536)	0.139
**Intensity-to-time curve**	Type I Type II Type III	2/73 21/73 50/73	0.245	0.268 (0.052–1.377) 1.093 (0.515–2.319) 1.250 (0.613–2.548)	0.115 0.817 0.539		
**ADC value**	Very low Low Intermediate	41/60 18/60 1/60	0.036^a^	2.937 (1.385–6.228) 0.339 (0.159–0.723) 0.873 (0.053–14.29)	0.005 0.005 0.924	2.191 (0.806–5.951)	0.124
**Extent of disease**	Unifocal Multifocal Multicentric	29/73 27/73 17/73	0.011	0.494 (0.249–0.980) 3.522 (1.504–8.246) 0.759 (0.351–1.640)	0.044 0.004 0.483	2.247 (0.771–6.548)	0.138

^a^Bonferroni’s post hoc analysis found no significance. ^b^*OR* Odds ratio, *CI* Confidence interval,  *ADC* Apparent diffusion coefficient

### Tumour size

**Fig. 2:**
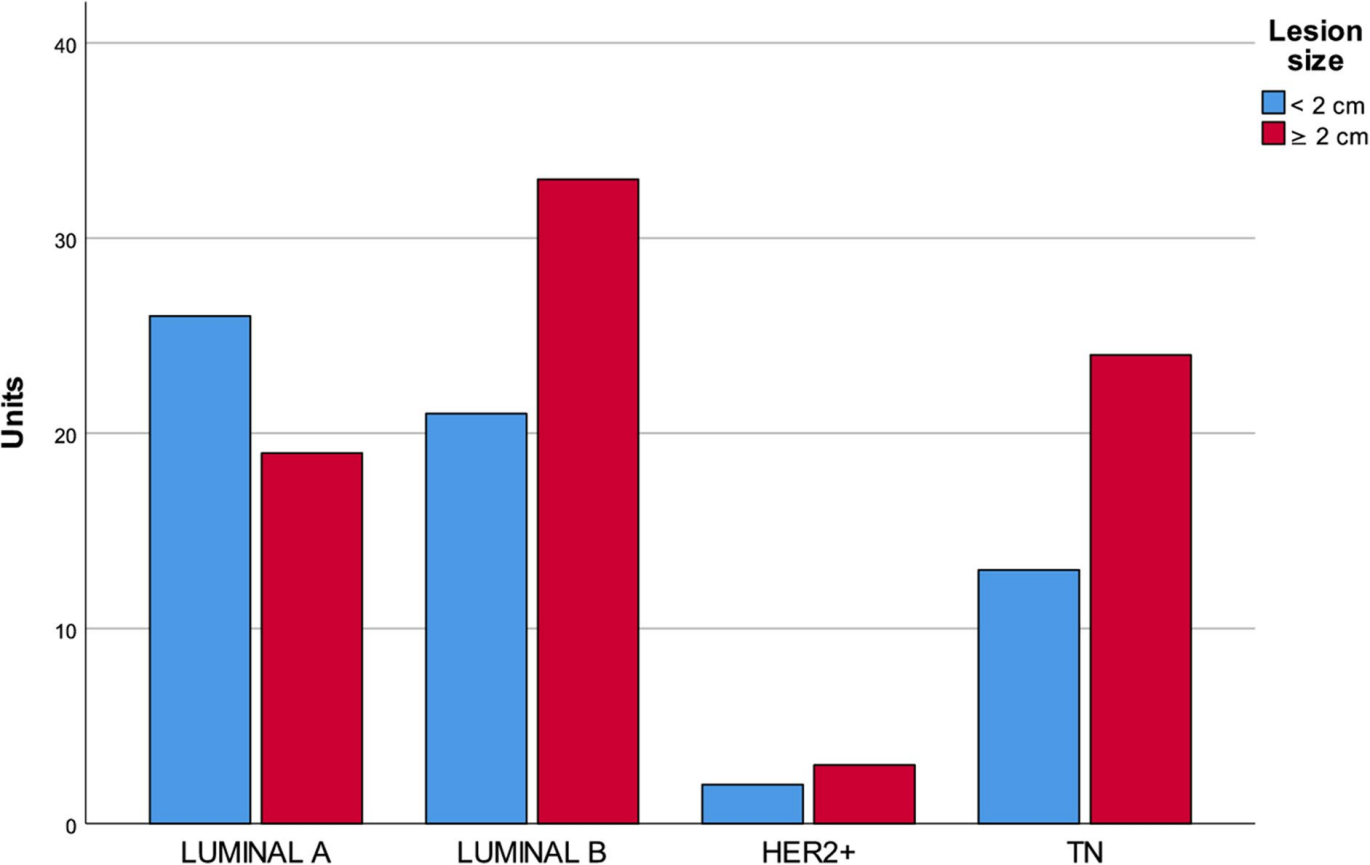
Distribution of the molecular subtypes of breast cancer (luminal A-like, luminal B-like, HER2 positive, and triple negative) according to lesions size

Median invasive BCs size at MR imaging was 53 mm (range 6–100 mm); 44% of the lesions were < 2 cm in size, while 56% were ≥ 2 cm. There was a significant association between lesion size < 2 cm and luminal A-like tumours. No statistical association was found between tumour size and the other molecular subtypes, even if luminal B and triple negatives resulted to be larger than the others (Fig. 2).

### Luminal A-like tumours

**Fig. 3:**
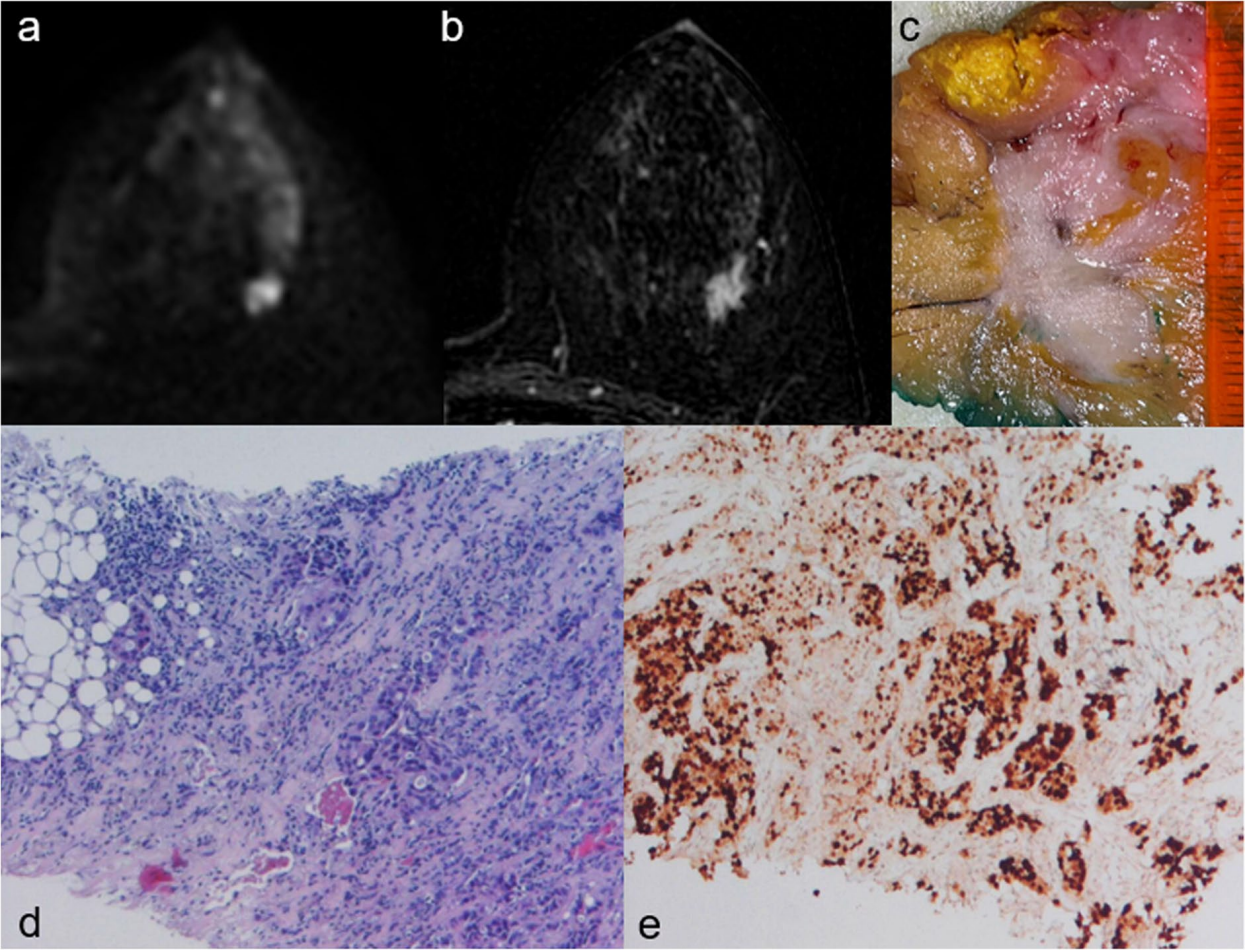
A 42-year-old woman with a luminal A-like carcinoma in the outer upper quadrant of the left breast. **a** Axial diffusion-weighted image (*b*-value = 1,000 s/mm.^2^) shows the high signal intensity of the mass, corresponding to a restricted diffusion area. **b** Axial post-contrast T1-weighted subtracted image shows a 14-mm mass with irregular shape, spiculated margins, and heterogeneous enhancement. **c** On cut surface, the lesion has irregular and infiltrative borders. **d**–**e** Histologic examination confirms the diagnosis of infiltrating carcinoma of “no special type” with diffuse expression of oestrogen receptor on immunohistochemistry

Most luminal A-like tumours were mass lesions (77.8%) characterised by irregular shape (42.9%), non-circumscribed margins (94.3%) and the absence of rim enhancement (91.4%) (Fig. 3). Statistical analyses showed a significant association between luminal A-like status and the absence of rim enhancement, of intralesional necrosis, of peritumoural oedema and of axillary adenopathy. The univariate regression analysis showed that lesion size ≥ 2 cm, rim enhancement, intralesional necrosis, peritumoural oedema and axillary pathological lymph nodes were negative predictors of luminal A-like cancers. The multivariate analysis confirmed that the absence of rim enhancement was independently associated with luminal A-like lesions (Table 3).

**Table 3:** Association between luminal A-like breast cancer and magnetic resonance imaging features

		**Luminal A**	**χ2** ***p*-value**	**Univariate analysis**		**Multivariate analysis**	
				**OR (CI 95%)**^a^	***p*-value**	**OR (CI 95%)**^a^	***p*-value**
**No. of patients**		45					
**Tumour size**	Median (mm) Range (mm)	18 6–100					
** ≥ 2 cm**		19/45	0.024	0.438 (0.213–0.902)	0.025	0.411 (0.151–1.118)	0.082
**T2 intensity**	Hypointensity Isointensity Hyperintensity	20/45 12/45 13/45	0.941	0.945 (0.464–1.927) 1.154 (0.513–2.595) 0.939 (0.431–2.043)	0.877 0.729 0.873		
**Mass enhancement**		35/45	0.525	0.753 (0.313–1.810)	0.526		
**Rim enhancement**		3/35	< 0.001	0.101 (0.029–0.358)	< 0.001	0.163 (0.040–0.657)	0.011
**Intralesional necrosis**		10/45	0.001	0.252 (0.112–0.566)	0.001	0.711 (0.228–2.220)	0.557
**Perilesional** **oedema**		12/45	< 0.001	0.199 (0.091–0.436)	< 0.001	0.419 (0.149–1.175)	0.098
**Abnormal lymph nodes**		10/45	0.01	0.352 (0.157–0.791)	0.012	0.600 (0.212–1.693)	0.334
**Intensity-to-time curve**	Type I Type II Type III	4/45 11/45 30/45	0.977	1.073 (0.306–3.769) 0.919 (0.405–2.083) 1.048 (0.495–2.216)	0.912 0.839 0.903		
**ADC value**	Very low Low Intermediate	20/40 19/40 1/40	0.182	0.491 (0.228–1.058) 1.944 (0.899–4.200) 2.154 (0.131–35.34)	0.069 0.091 0.591		
**Extent of disease**	Unifocal Multifocal Multicentric	19/45 10/45 16/45	0.148	0.828 (0.405–1.692) 0.599 (0.263–1.364) 2.097 (0.957–4.593)	0.605 0.222 0.064		

^a^*OR* Odds ratio, *CI* Confidence interval, *ADC* Apparent diffusion coefficient

### Luminal B-like tumours

Most luminal B-like were mass lesions (81.5%), characterised by round shape (56.8%), non-circumscribed margins (77.3%) and the absence of rim enhancement (63.7%). *χ*^2^ analyses showed an inverse association between luminal B-like tumours and multicentric disease. Regression analyses proved that multicentric disease is a negative predictor of luminal B status (Table 4).

**Table 4:** Correlation between luminal B-like tumours and MRI features

		**Luminal B**	**χ2 analysis *p*-value**	**Univariate analysis**	
				**OR (CI 95%)**^a^	***p*-value**
**No. of patients**		54			
**Tumour size**	Median (mm) Range (mm)	20 6–86			
** ≥ 2 cm**		33/54	0.338	1.401 (0.702–2.793)	0.339
**T2 intensity**	Hypointensity Isointensity Hyperintensity	29/54 9/54 16/54	0.159	1.723 (0.868–3.420) 0.469 (0.201–1.098) 0.988 (0.470–2.076)	0.120 0.081 0.974
**Mass enhancement**		44/54	0.881	1.069 (0.449–2.544)	0.881
**Rim enhancement**		16/44	0.944	1.029 (0.469–2.255)	0.944
**Intralesional necrosis**		23/54	0.899	0.957 (0.482–1.900)	0.899
**Perilesional oedema**		32/54	0.204	1.558 (0.784–3.097)	0.205
**Abnormal lymph nodes**		23/54	0.334	1.410 (0.702–2.831)	0.335
**Intensity-to-time curve**	Type I Type II Type III	5/54 14/54 35/54	0.961	1.166 (0.351–3.877) 1.034 (0.475–2.250) 0.921 (0.451–1.882)	0.802 0.933 0.822
**ADC value**	Very low Low Intermediate	29/48 19/48 0/48	0.490	0.922 (0.440–1.932) 1.213 (0.576–2.554) Out of scale	0.830 0.611
**Extent of disease**	Unifocal Multifocal Multicentric	28/54 19/54 7/54	0.025	1.526 (0.770–3.022) 1.604 (0.766–3.357) 0.298 (0.120–0.740)	0.226 0.210 0.009

^a^*OR* Odds ratio, *CI* Confidence interval, *ADC* Apparent diffusion coefficient

#### HER2-positive tumours

All the 5 HER2-positive tumours included in our study population were masses characterised by non-circumscribed margins, heterogeneous enhancement and without peritumoural oedema. Only one of them (20.0%) showed intralesional necrosis. *χ*^2^ analysis showed an inverse association between HER2-positive cancers and peritumoural oedema, although regression analyses showed no significant predictors (Table 5).

**Table 5:** Correlation between HER2-positive tumours and magnetic resonance imaging features

		**HER2 + **	**χ2 analysis *p*-value**	**Univariate analysis**	
				**OR (CI 95%)**^a^	***p*-value**
**No. of patients**		5			
**Tumour size**	Median (mm) Range (mm)	25 12–36			
** ≥ 2 cm**		3/5	0.885	1.184 (0.192–7.316)	0.856
**T2 intensity**	Hypointensity Isointensity Hyperintensity	3/5 2/5 0/5	0.322	1.844 (0.299–11.39) 2.081 (0.333–12.99) Out of scale	0.510 0.433
**Mass enhancement**		5/5	0.268	Out of scale	
**Rim enhancement**		0/5	0.087	Out of scale	
**Intralesional necrosis**		1/5	0.285	0.317 (0.034–2.908)	0.309
**Perilesional oedema**		0/5	0.017	Out of scale	
**Abnormal lymph nodes**		2/5	0.910	1.111 (0.180–6.875)	0.910
**Intensity-to-time curve**	Type I Type II Type III	0/5 1/5 4/5	0.720	Out of scale 0.721 (0.078–6.674) 2.112 (0.230–19.442)	0.774 0.509
**ADC value**	Very low Low Intermediate	4/5 1/5 0/5	0.681	2.575 (0.279–23.75) 0.417 (0.045–3.845) Out of scale	0.404 0.440
**Extent of disease**	Unifocal Multifocal Multicentric	2/5 2/5 1/5	0.857	0.796 (0.129–4.915) 1.658 (0.267–10.31) 0.721 (0.078–6.674)	0.806 0.588 0.774

^a^*OR* Odds ratio, *CI* Confidence interval, *ADC* Apparent diffusion coefficient

#### Triple negative tumours

**Fig. 4:**
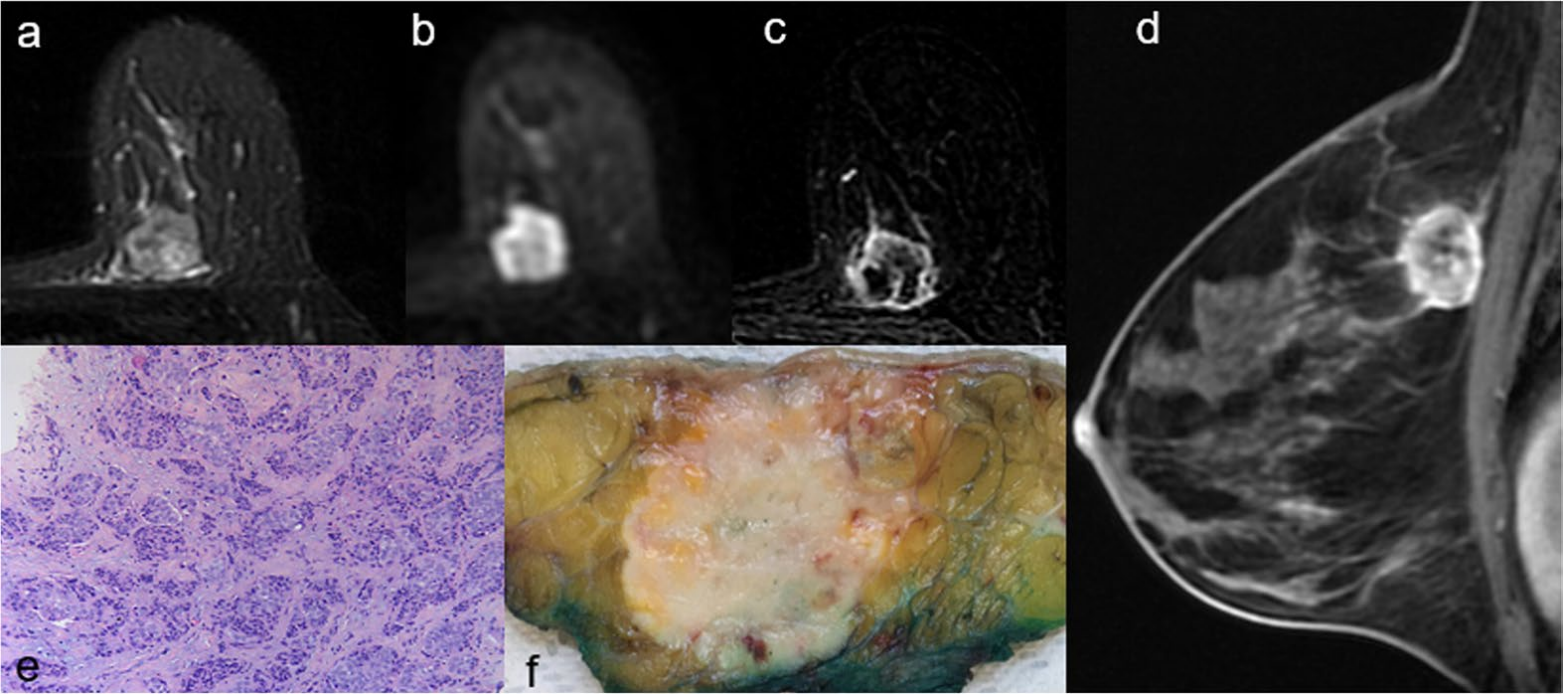
A 45-year-old woman with triple-negative tumour of the left breast. **a** Axial fat-suppressed T2-weighted image shows an 18-mm, heterogeneously hyperintense round mass with non-circumscribed margins in the upper inner quadrant of the left breast. **b** Axial diffusion-weighted imaging (*b*-value = 1,000 s/mm.^2^) shows peripheral high signal intensity and central hypointensity. **c** Axial and (**d**) sagittal post-contrast T1-weighted subtracted images show a corresponding irregular round mass with rim enhancement. **e** On cut surface, the lesion is round shaped with smooth margins. **f** Histologic examination confirms the diagnosis of infiltrating carcinoma of “no special type”

Most triple negative tumours were masses (81.1%) characterised by round shape (86.7%), non-circumscribed margins (90%) and rim enhancement (73.3%) (Fig. 4). Statistical analyses showed a significant association between triple-negative tumours and round shape, rim enhancement, intralesional necrosis and the presence of peritumoural oedema (Table 6). The univariate regression analysis proved that round shape in mass lesions, rim enhancement, intralesional necrosis and peritumoural oedema were predictors of triple negative lesions, while low ADC value was a negative predictor. The multivariate analysis confirmed that round shape in mass lesions, rim enhancement, and peritumoural oedema were associated with triple negatives (Table 6).

**Table 6:** Correlation between triple-negative tumours and magnetic resonance imaging features

		**Triple negative**	**χ2 analysis** ***p*-value**	**Univariate analysis**		**Multivariate analysis**	
				**OR (CI 95%)**^a^	***p*-value**	**OR (CI 95%)**^a^	***p*-value**
**No. of** **patients**		37					
**Tumour size**	Median (mm) Range (mm)	23 9–90					
** ≥ 2 cm**		24/37	0.207	1.645 (0.756–3.577)	0.209		
**T2 intensity**	Hypointensity Isointensity Hyperintensity	12/37 12/37 13/37	0.173	0.480 (0.218–1.056) 1.690 (0.737–3.875) 1.401 (0.630–3.116)	0.068 0.215 0.409		
**Mass enhancement**		30/37	0.967	1.020 (0.392–2.655)	0.967		
**Shape** **(Mass)**	Round Oval Irregular	26/30 0/30 4/30	0.001	7.150 (2.295–22.28) Out of scale 0.205 (0.066–0.640)	0.001 0.006	5.319 (1.425–19.85)	0.013
**Rim** **enhancement**		22/30	< 0.001	9.408 (3.613–24.50)	< 0.001	9.155 (2.537–33.04)	0.001
**Intralesional necrosis**		27/37	< 0.001	5.559 (2.416–12.79)	< 0.001	0.846 (0.214–3.340)	0.811
**Perilesional** **oedema**		30/37	< 0.001	5.844 (2.352–14.52)	< 0.001	3.852 (1.092–13.59)	0.036
**Abnormal lymph nodes**		18/37	0.106	1.868 (0.871–4.003)	0.108		
**Intensity-to-time curve**	Type I Type II Type III	3/37 10/37 24/37	0.969	0.931 (0.238–3.644) 1.111 (0.475–2.601 0.936 (0.426–2.059)	0.919 0.808 0.870		
**ADC value**	Very low Low Intermediate	24/32 7/32 1/32	0.106	2.264 (0.921–5.565) 0.388 (0.152–0.986) 2.968 (0.180–48.88)	0.075 0.047 0.447	4.762 (0.018–1277)	0.584
**Extent of disease**	Unifocal Multifocal Multicentric	15/37 10/37 12/37	0.530	0.765 (0.358–1.638) 0.872 (0.377–2.017) 1.600 (0.701–3.654)	0.491 0.749 0.265		

^a^*OR* Odds ratio, *CI* Confidence interval, *ADC* Apparent diffusion coefficient

## Discussion

BC is a heterogeneous disease, with distinct molecular subtypes which have both prognostic and predictive value. In this context, precision medicine involves the use of biomarkers to create customised treatments. Furthermore, the possibility to draw a reliable correlation between molecular subtypes and imaging features of BC is envisaged to improve patients’ care. As a consequence, nowadays, imaging aims to offer a complementary, noninvasive method to obtain biological information about BC, in addition to traditional tissue-sampling-derived biomarkers.

Breast MRI is considered the more promising technique to differentiate tumour subtypes noninvasively [6, 16]. Contrast-enhanced sequences are the backbone of any breast MRI protocol, providing information about morphological and kinetic features of BC. To overcome the suboptimal specificity of contrast-enhanced MRI, functional techniques, such as MR spectroscopy and DWI, have been widely investigated and progressively introduced into routine clinical practice. Nowadays, a basic mpMRI protocol includes unenhanced sequences (T2 weighted and DWI) followed by the series of pre- and post-contrast T1-weighted acquisitions [13], since it was demonstrated that mpMRI including contrast-enhanced sequences and DWI increases diagnostic accuracy in BC diagnosis [16, 24]. Also, magnetic resonance spectroscopy improves the diagnostic accuracy of breast MRI [25–29]; however, technical challenges and operator dependency have limited large-scale implementation of this technique [30].

The purpose of our study was to clarify whether diagnostic mpMRI at 3 T could be a reliable noninvasive predictor of histological tumour type and molecular subtype of BC. Most of the patients included in this study (78.2%) were affected by invasive carcinoma NST, which is the most common type of BC, 12% by ILC, and about 10% by DCIS. Our distribution substantially reflected data reported in literature [31–35].

The specific features of DCIS are calcifications at digital mammography (70–90% of cases [36]) and non-mass enhancement at MRI contrast-enhanced sequences (up to 81% of cases [34, 37–39]); our results (86.7% of DCIS presented as non-mass enhancements, *p* < 0.001) are in substantial agreement with those data. A second MRI feature significantly associated with DCIS was T2 isointensity (78.6%, *p* < 0.001) (Fig. 1), also in agreement with data reported in literature too [39]. DCIS was significantly associated with the absence of axillary adenopathy (0%, *p* = 0.003); this result was expected, since DCIS is confined to the mammary ductal lobular system, without invasion of the basement membrane. However, the occurrence of lymph node metastases is still possible (likely due to missing areas of microinvasion in large-sized tumours or iatrogenic dissemination of tumour cells during preoperative breast biopsy [36]).

The term invasive carcinoma NST identifies a subset of invasive BCs that cannot be classified morphologically as any of the special histological types. ILC, instead, is characterised by a typical discohesive morphology and by the loss of E-cadherin function. The small cohort of ILC included in the study (although reflecting data in literature [31–33]) could explain the lack of statistical significance of our results. On the contrary, MRI features significantly associated with invasive carcinomas NST were as follows: mass enhancement, intralesional necrosis and abnormal axillary lymph nodes. The majority of invasive BCs appear as masses with intermediate to low signal intensity on T2-weighted images, due to high cellularity and low water content [13]; our results are in line with these findings. The contemporary association between invasive BC of NST and intralesional necrosis (that shows high signal on T2-weighted images by definition [7]) can be explained by the fact that the evaluation of T2-signal intensity in this study was based on the predominant signal intensity of each lesion; therefore, lesions with only small areas of intralesional necrosis were still classified as isointense or hypointense.

As expected, rim enhancement, intralesional necrosis, peritumoural oedema and the presence of metastatic axillary lymph nodes were predictors of G3 status. These results emphasise the evidence that poorly differentiated, aggressive BCs are associated with poor prognostic indicators at breast MRI. Moreover, we showed an inverse correlation between tumour grading and ADC values, since very low ADC values were significantly associated with high tumour grades. This result has confirmed the predicting value of DWI regarding nuclear grading, as suggested by a few previous studies [10, 11, 17].

The vast majority of invasive breast lesions included in this study were luminal-like (70.2%), while triple-negative and HER2-positive cases represented a minority of the sample (26.2% and 3.6%, respectively). These data reflect the lower frequency of these molecular subtypes and are comparable to similar series in literature [40, 41]. In this study, luminal-like and HER2-positive BCs were more frequently characterised by irregular shape. In particular, most luminal A-like were irregular-shaped (42.9%) masses (77.8%), with non-circumscribed margins (94.3%) and without rim enhancement (91.4%).

Furthermore, our study has demonstrated that the absence of MRI features on T2-weighted images associated with poor prognosis, such as intralesional necrosis [42] and peritumoural oedema [43, 44], was significantly associated with luminal A-like tumours (Fig. 3). In particular, the absence of peritumoural oedema resulted independently associated with the luminal A-like status, in agreement with previous studies [8, 10, 45]. Finally, luminal A-like tumours in our study were significantly associated with the absence of axillary adenopathy, confirming that this BC subtype is characterised by a less aggressive behaviour and a better prognosis [46].

Triple negative BCs are biologically and clinically aggressive tumours with peculiar imaging features, on both conventional breast imaging (frequently mimicking benign lesions [47–49]) and MRI. In our study, triple negative BCs were predominantly masses (81.1%), characterised by round shape (86.7%), non-circumscribed margins (90%) and rim enhancement (73.3%). Round shape and rim enhancement were independently associated with the triple-negative status (Fig. 4). These data confirm existing evidence in literature [7, 9, 10, 45, 50–54]. The typical regular shape can be explained by the frequent occurrence of “pushing”, non-infiltrative growth pattern of triple negatives compared to other subtypes of BC, while the presence of rim enhancement on contrast-enhanced sequences has been associated with increased angiogenesis and vascular endothelial growth factor expression and with the lack of oestrogen and progesterone receptors [55, 56]. Among MRI features on T2-weighted images, intralesional necrosis and peritumoural oedema have proved to be positive predictor of the triple-negative status. These results are in accordance with previous literature [7, 8, 10, 45, 50, 53].

Our study has some limitations. First, it was a single-centre, retrospective study, and the cohort of patients enrolled was relatively small, and the number of triple-negative and HER2-positive BCs was even smaller, because those patients tend to receive neoadjuvant chemotherapy. Secondly, MRI datasets were evaluated by two readers, in consensus, not considering interobserver variability. In addition, due to technical problems, percutaneous biopsies under mammographic guidance were not performed during the enrolment period, and this could have caused an underestimation of the number of DCIS cases. Finally, molecular subtypes were determined using immunohistochemical surrogates, which lack in standardisation compared to gene profiling, even if they have shown similar clinical significance and are nowadays routinely used [4].

In conclusion, we showed that mpMRI at 3-T MRI has proved to be a valid noninvasive tool to distinguish between BC subtypes, especially luminal A-like and triple negative, even though histopathology remains the standard of care also in the present time of rapid development of advanced breast imaging.

## Data Availability

The datasets generated during and/or analysed during the current study are available from the corresponding author on reasonable request.

## Abbreviations

ADC: Apparent diffusion coefficient
BC: Breast cancer
DCIS: Ductal carcinoma *in situ*
DWI: Diffusion-weighted imaging
ET: Echo-time
FOV: Field of view
HER2: Human epidermal growth factor receptor 2
ILC: Invasive lobular carcinoma
Ki-67: Proliferation index
mpMRI: Multiparametric magnetic resonance imaging
NEX: Number of excitations
NST: No special type
RT: Repetition time
